# Notes and News

**Published:** 1903-02-28

**Authors:** 


### NOTES AND NEWS.

The Yarrow Convalescent Home for Children intimate that from Thursday, February 26th, their offices will be removed to Terminus Chambers, 6 Holborn Viaduct, E.C.

Sir Frederick Treves opened the new operating theatre at the Dorset County Hospital this week, to which he has presented an operation table of the most approved pattern.

Mr. H. R. S. Druce, who for the last six years has been on the secretarial staff of the Dreadnought Hospital, Greenwich, has been appointed secretary to the Central London Ophthalmic Hospital. Mr. Druce is a solicitor by profession, and has taken much interest in hospital work.

The Spanish Government is taking vigorous measures to combat tuberculosis. Special sums are to be set apart yearly for the establishment and maintenance of sanatoria for the use of poor patients. The Public Health Department is further elaborating a social scheme of defence against and prevention of the spread of the disease.

An inquiry was held with reference to the proposed new scheme for the administration of Addenbrooke's Hospital, Cambridge, by the Charity Commissioners through their assistant commissioner, Mr. Henry Bowyear. The assistant commissioner, on closing the inquiry, said that he was afraid that probably the scheme would not be ready for Parliament in time, owing to the difficulties introduced by the number of objections brought forward.

A meeting of the Hospitals Association will be held on Tuesday, March 3rd, at the Caxton Hall, Westminster, at 5 p.m., Sir Trevor Lawrence, treasurer of St. Bartholomew's Hospital, in the chair. Sir Henry Burdett will open a discussion on "Hospital Sites and Population in London" to consider the questions: "Ought any, and if so, which of the large hospitals to be moved from their present sites to new ones situated either in more congested districts or in the country." Admission tickets can be obtained from Mr. Sydney Phillips, St. Thomas's Hospital.

It is reported that a new enemy to the malaria mosquito has been discovered by Dr. Dempwolff, who has been pursuing investigations in German New Guinea. This is an aquatic insect which stings the mosquito; and it is hoped that this insect can be used as a means of exterminating the anopheles mosquito in various districts.

It is evident that Sheffield means to fight hard for its proposed position as the Yorkshire University. It appears that a plan to amalgamate the universities of Leeds and Sheffield would find support, but if only one university can represent Yorkshire, then Sheffield will urge its claims before those of Leeds. It holds that it possesses a medical department well up to date and suited for a progressive and growing medical school.

The site for the King's Sanatorium has been chosen. It is situated in one of the healthiest and most beautiful parts of England, near Hindhead Common, in the Haslemere District. The extent of the site is 125 acres, and we learn that it is proposed to erect the Sanatorium for 100 beds, and that part of these will be reserved for well-to-do patients paying as much as five guineas weekly. We hope that the fact will not be lost sight of that there is not much difficulty in securing accommodation for either the very rich or the very poor. It is the patient who can pay from fifteen shillings to two guineas who is the worst provided for at present.

The National Canine Defence League is preparing petitions for presentation to Parliament protesting against the imposition of the muzzling order in certain Welsh counties, quarantine regulations and isolation, and departmental orders restricting the movement of dogs into or out of certain districts, and ordaining the manner in which they shall be kept. The league requires of its members "not to vote for any Parliamentary candidate who endorses the rabies returns (which are held to be utterly fallacious) of the Board of Agriculture, and supports the methods of muzzle and slaughter employed by that Board," and asks them to sign another which runs "On account of the terrible cruelties perpetrated on living animals in vivisectional laboratories and in Pasteur and preventive medicine institutes, I hereby undertake not to employ as medical attendant anyone who practises vivisection anywhere in any form."

The last mentioned pledge will not create that profound alarm which those who framed it in their ignorance probably imagined. How many general practitioners are there who practise vivisection? The ignorant public who sign such documents probably imagine the family doctor torturing dogs and cats for his amusement in his few leisure moments. They know nothing of the laws restricting the practice of vivisection, still less do they regard with an alarm they might reasonably feel, the reposeful manner in which some members of the profession rest on the knowledge acquired in the schools, and show no interest in the advances in the great battlefield against disease, or any inclination to enter into laborious investigations in the pursuit of which vivisection might be necessary.

